# Efficacy and safety of telitacicept combined with immunosuppressive therapy for IgA nephropathy: a retrospective multicenter cohort study

**DOI:** 10.3389/fimmu.2026.1740891

**Published:** 2026-02-11

**Authors:** Fang Zeng, Yang Yang, Fei Tan, Yebei Li, Daijin Ren, Chengmin Cai, Jiang Wang, Wenjun Yan, Xiaojie Xie, Yu Wang, Lu Yi, Shizhang Xu, Dehui Liu, Gaosi Xu

**Affiliations:** 1 Department of Nephrology, The Second Affiliated Hospital, Jiangxi Medical College, Nanchang University, Nanchang, China; 2 Department of Nephrology, The Affiliated Ganzhou Hospital, Jiangxi Medical College, Nanchang University, Ganzhou, China; 3 Department of Health Management Center, The First Affiliated Hospital, Jiangxi Medical College, Nanchang University, Nanchang, China; 4 Department of Health Management Center, Jiangxi Provincial People’s Hospital, Nanchang, China; 5 Department of Nephrology, Jiujiang Hospital of Traditional Chinese Medicine, Jiujiang, China; 6 Department of Nephrology, The Second Affiliated Hospital of Jiujiang University, Jiujiang, China; 7 Department of Nephrology, The First Affiliated Hospital of Gannan Medical University, Ganzhou, China; 8 Department of Nephrology, Yingtan 184 Hospital, Yingtan, China; 9 Department of Nephrology, Yichun People’ s Hospital, Yichun, China

**Keywords:** EGFR, hematuria, IgA nephropathy, proteinuria, telitacicept

## Abstract

**Background:**

To investigate the efficacy and safety of telitacicept combined with glucocorticoid/mycophenolate mofetil (GM) versus telitacicept in the treatment of immunoglobulin A nephropathy (IgAN).

**Methods:**

This retrospective, multicenter cohort study enrolled patients aged ≥ 18 years with biopsy-proven IgAN who were treated with telitacicept. All participants had a baseline eGFR ≥ 30 mL/min/1.73 m² and proteinuria excretion ≥ 0.75 g/day after receiving at least 1 month of optimized supportive treatment. Clinical data were collected over 9 months.

**Results:**

A total of 256 patients were enrolled in the study, with 125 patients receiving telitacicept monotherapy and 131 patients receiving telitacicept plus GM therapy. The mean age was 39.5 ± 11.6 years, and 100 patients (39.1%) were male. At 9 months, the telitacicept + GM group showed a significantly greater reduction in proteinuria (-1.32 g/day versus -0.94 g/day, *P* < 0.001) and exhibited better eGFR slope (2.74 mL/min/1.73 m^2^/year versus -0.56 mL/min/1.73 m^2^/year, *P* = 0.014) compared to the telitacicept group. Similar reductions in urinary red blood cell count were observed in both groups, but the telitacicept + GM group exhibited a significantly greater median change at 9 months (-35.5/µL versus -9.0/µL, *P* = 0.009). Kaplan-Meier analysis revealed a significant benefit for the telitacicept + GM group over telitacicept monotherapy in both complete (54.2% vs. 40.8%, *P* = 0.005) and overall (78.6% vs. 70.4%, *P* = 0.014) remission rates. After adjusting for various factors, multivariable Cox regression analysis indicated a significantly higher likelihood of complete [adjusted HR 2.18 (95% CI 1.46-3.22), *P* < 0.001] and overall [adjusted HR 1.46 (95% CI 1.08-2.00), *P* = 0.013] remissions in the telitacicept + GM group at 9 months. Benefits were consistent across subgroups, with no significant interaction effects. Although the telitacicept + GM group was associated with a higher overall incidence of adverse events (28.2% versus 11.2%), no serious adverse events were reported in either group.

**Conclusion:**

Telitacicept + GM regimen significantly reduced proteinuria and hematuria, maintained stable renal function in patients with IgAN, with no serious adverse events occurring.

## 1 Introduction

Immunoglobulin A nephropathy (IgAN) is the most prevalent primary glomerulonephritis globally, carrying a high lifetime risk of renal failure and an enormous socioeconomic burden (1). Even among patients with urinary protein controlled within 1.0 g/d, up to 22%-30% still progress to end-stage renal disease (ESRD) within 10 years (2, 3). With growing insights into the ‘four-hit theory’ of IgAN pathogenesis (4), an increasing number of drugs, including budesonide (5), telitacicept (6), felzartamab (7), and empagliflozin (8), which target the immune components of IgAN, have been identified and appear promising. Among these strategies, targeting B-cell activating factor (BAFF) and a proliferation-inducing ligand (APRIL) blockade is considered a potentially effective approach for managing IgAN (9).

Telitacicept, a dual blocker of BAFF and APRIL, reduces autoreactive B-cell populations and the generation of aberrant IgA1 antibodies (10). The phase 2 clinical trial of telitacicept for IgAN demonstrated that it significantly reduced proteinuria compared to placebo at 24 weeks (11). Subsequent real-world studies have corroborated this evidence, demonstrating that telitacicept achieves a median proteinuria reduction ranging from 28.6% to 66.8%, accompanied by renal function stabilization and a favorable safety profile (12–14). However, the reduction in proteinuria achieved with telitacicept is not enough to satisfy clinical requirements, leaving certain patients at a high risk of disease progression. Therefore, combination therapies are needed.

The reduced-dose systemic glucocorticoid regimen, as established by the TESTING study for progressive IgAN (15, 16), has been adopted and is recommended by the latest KDIGO guideline (17). As a glucocorticoid-sparing agent, mycophenolate mofetil (MMF) reduces B- and T-cell proliferation and antibody production, and is widely used in China for treating IgAN (18, 19). Prompted by the potential for synergy, we conducted this retrospective, real-world study to compare the efficacy and safety of telitacicept combined with immunosuppressive therapy versus telitacicept monotherapy in patients with IgAN.

## 2 Methods

### 2.1 Study design and population

The retrospective, multicenter cohort study was conducted across 5 centers in Jiangxi Province, China, from April 2023 to December 2024. Inclusion criteria were: (1) age between 18 and 65 years; (2) biopsy-proven primary IgAN; (3) patients treated with 160 mg telitacicept weekly; (4) proteinuria excretion ≥ 0.75 g/day after receiving stable and optimized renin-angiotensin-aldosterone system inhibitor therapy for ≥ 1 months; (5) estimated glomerular filtration rate (eGFR) ≥ 30 mL/min/1.73m^2^ (calculated via the Chronic Kidney Disease Epidemiology Collaboration creatinine equation (20)). Exclusion criteria were: (1) secondary IgAN, including but not limited to Henoch-Schönlein purpura, systemic lupus erythematosus, viral hepatitis, rheumatoid arthritis and psoriasis; (2) lack of follow-up data. This study received approval from the Ethics Committee of the Second Affiliated Hospital of Nanchang University (Approval No. IIT-O-2025-270).

### 2.2 Treatment regimen

Participants were categorized into two groups based on their treatment regimens: the telitacicept group and the telitacicept + glucocorticoid/mycophenolate mofetil (GM) group. All patients received subcutaneous telitacicept at 160 mg weekly for 6 months, followed by a reduced frequency of 160 mg every other week for an additional 3 months. Hepatitis B carriers concurrently received ongoing antiviral prophylaxis. Patients in the telitacicept + GM group additionally received low-dose prednisone (0.4-0.6 mg/kg/day for 1 month, tapered by 20% every 2 weeks) or MMF (1.0-1.5 g/day during follow-up). Prophylaxis against Pneumocystis jirovecii pneumonia, as well as gastroprotection and bone protection, were also provided concomitantly in the telitacicept + GM group.

### 2.3 Study outcomes

The primary outcomes were the changes in 24-hour proteinuria and eGFR. The secondary outcomes included remission rate, urinary red blood cells (RBC) count, serum albumin, lymphocyte subset cells and immunoglobulin levels. Complete remission (CR) was defined as 24-hour proteinuria ≤ 0.3 g/d, serum albumin (Alb) > 35 g/L, and stable renal function, characterized by a decrease in eGFR of ≤ 30%. Partial remission (PR) was defined as a reduction in proteinuria exceeding 50% from baseline and stable renal function, while not fulfilling the criteria for CR. The overall remission (OR) rate was calculated as the sum of CR and PR.

### 2.4 Statistical analysis

Statistical analyses were conducted using SPSS version 25.0 (IBM, Armonk, NY, USA), GraphPad Prism version 10.6, and R version 4.5.1 software. Data are expressed as mean ± standard deviation for normally distributed continuous variables, while non-normally distributed continuous variables are summarized as median (interquartile range). Data from patients lost to follow-up were censored at their last visit, and multiple imputation was applied for covariates with missing values. Comparison between the two groups for these variables were performed using independent-samples t-test and the Mann-Whitney U-test, respectively. For the longitudinal assessment of proteinuria, eGFR, urinary RBC count, and serum albumin during follow-up periods (at 1, 2, 3, 4, 5, 6, and 9 months), these variables were log-transformed and analyzed using analysis of covariance with baseline adjustment to control for regression to the mean. A linear mixed effects model was used to calculate the eGFR slope for both groups over the entire intervention period. Categorical variables are presented as numbers (percentages) and were compared using the chi-squared test or Fisher’s exact test, as appropriate. Kaplan-Meier curves were constructed to estimate the cumulative incidence of CR and OR in each group. Subsequently, multivariable Cox proportional hazards models were employed with an entry criterion of *P* < 0.1 in univariate analysis or based on clinical relevance. Furthermore, subgroup analyses were conducted, and the results are presented in a forest plot. *P* values less than 0.05 were considered statistically significant.

## 3 Results

### 3.1 Enrollment and baseline characteristics

**Figure 1:**
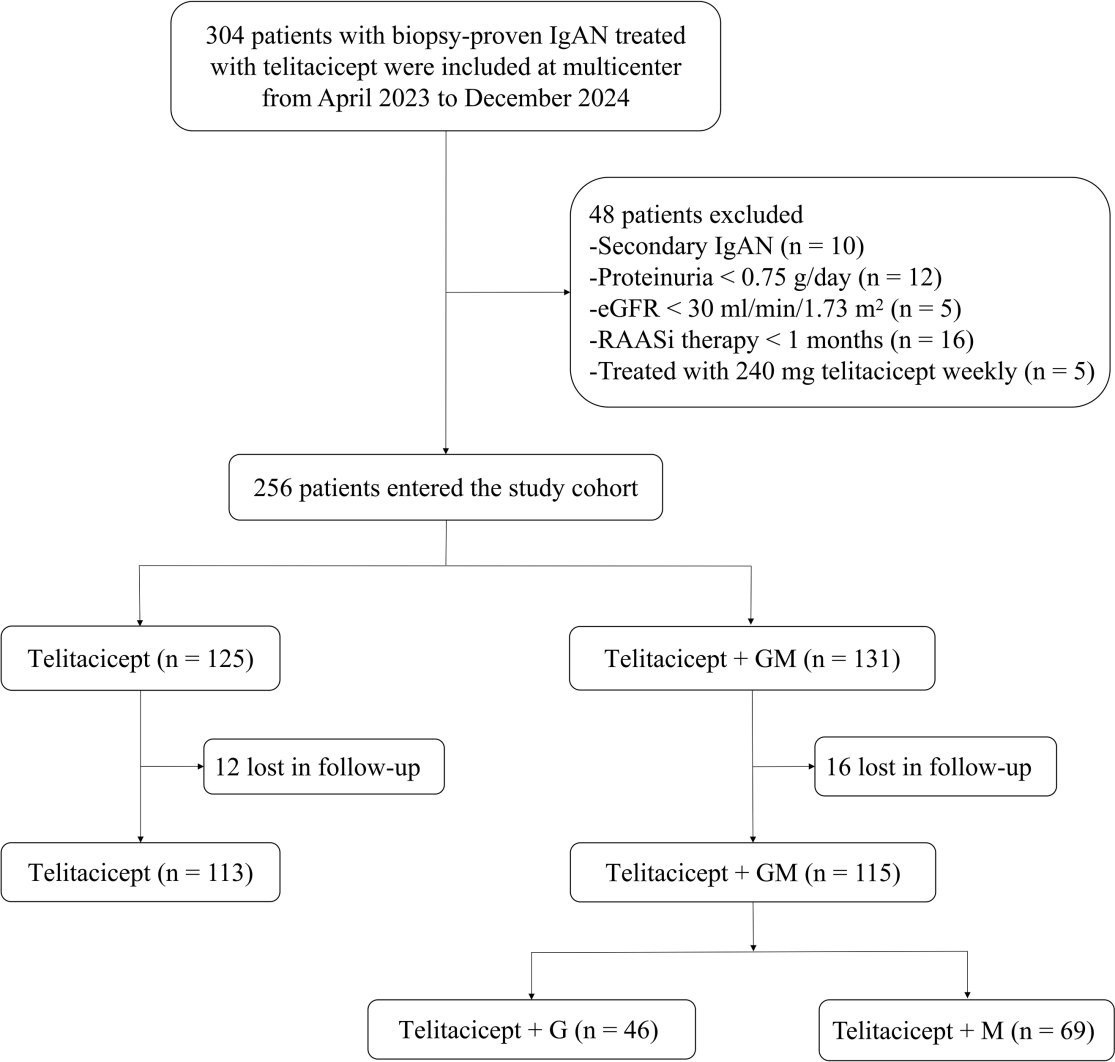
Flowchart of study design. IgAN, IgA nephropathy; eGFR, estimated glomerular filtration rate; RAASi: renin–angiotensin–aldosterone system inhibitor; GM, glucocorticoid/mycophenolate mofetil; G, glucocorticoid; M, mycophenolate mofetil.

A total of 256 patients with IgAN were included: 125 received telitacicept and 131 received telitacicept + GM (**Figure 1**). The mean age of the patients was 39.5 ± 11.6 years, and 100 (39.1%) of the patients were male. Baseline characteristics were well matched between the two groups, with no statistically significant differences observed in age, sex, 24-hour proteinuria, urinary RBC count, eGFR, serum albumin, and pathological features (**Table 1**).

**Table 1:** Baseline clinical and pathological characteristics of patients.

Variables	Telitacicept + GM (n = 131)	Telitacicept (n = 125)	*P*-value
Age (years)	38.6 ± 12.0	40.4 ± 11.2	0.215
Male, n (%)	46 (35.1)	54 (43.2)	0.185
BMI (kg/m^2^)	23.6 ± 3.7	23.6 ± 3.5	0.965
Hypertension, n (%)	65 (49.6)	71 (56.8)	0.250
SBP	131.9 ± 18.8	129.2 ± 19.0	0.257
DBP	85.1 ± 14.5	84.7 ± 12.4	0.872
24-hour proteinuria (g/day)	1.6 (1.1, 2.9)	1.5 (1.0, 2.0)	0.060
Urinary RBC count (cells/µL)	50.0 (10.0, 124.0)	28.0 (11.0, 77.0)	0.078
Hemoglobin (g/L)	126.2 ± 19.3	129.8 ± 19.4	0.146
Serum albumin (g/L)	39.5 ± 5.0	39.3 ± 4.2	0.704
Urea creatinine (µmol/L)	121.1 ± 66.3	131.9 ± 65.9	0.190
eGFR (mL/min/1.73 m^2^)	66.9 (41.5, 88.9)	57.2 (38.3, 80.8)	0.087
Serum uric acid (µmol/L)	383.1 ± 106.1	393.6 ± 121.7	0.460
ALT (U/L)	22.9 ± 19.5	20.6 ± 16.0	0.278
AST (U/L)	22.4 ± 12.2	21.8 ± 14.0	0.712
TC (mmol/L)	5.3 ± 1.3	5.3 ± 1.4	0.942
TG (mmol/L)	2.2 ± 1.6	2.3 ± 1.9	0.560
Glucose (mmol/L)	5.3 ± 1.1	5.2 ± 1.0	0.318
Serum IgG (g/L)	11.0 (9.0, 13.1)	11.4 (9.3, 13.2)	0.475
Serum IgA (g/L)	2.9 (2.2, 3.8)	2.9 (2.1, 3.8)	0.843
Serum IgM (g/L)	1.3 (0.9, 1.8)	1.1 (0.8, 1.5)	0.075
Oxford classification ^a^			
M1	109 (93.2)	98 (94.2)	0.745
E1	39 (33.3)	27 (26.0)	0.232
S1	79 (67.5)	67 (64.4)	0.627
T 1/2	56 (47.9)	60 (57.7)	0.144
C 1/2	58 (49.6)	41 (39.4)	0.130
Lee’s classification ^a^			0.493
II	15 (12.8)	18 (17.3)	
III	52 (44.4)	37 (35.6)	
IV	43 (36.8)	40 (38.5)	
V	7 (6.0)	9 (8.7)	

a Oxford classification and Lee’s classification was collected from 221 patients; GM, glucocorticoid/mycophenolate mofetil; BMI, body mass index; SBP, systolic blood pressure; DBP, diastolic blood pressure; RBC, red blood cells; eGFR, estimated glomerular filtration rate; ALT, alanine aminotransferase; AST, aspartate aminotransferase; TC, total cholesterol; TG, triglyceride; M, mesangial hypercellularity; E, endocapillary hypercellularity; S, segmental glomerulosclerosis; T, tubular atrophy/interstitial fibrosis; C, crescents.

### 3.2 Primary outcomes

**Figure 2:**
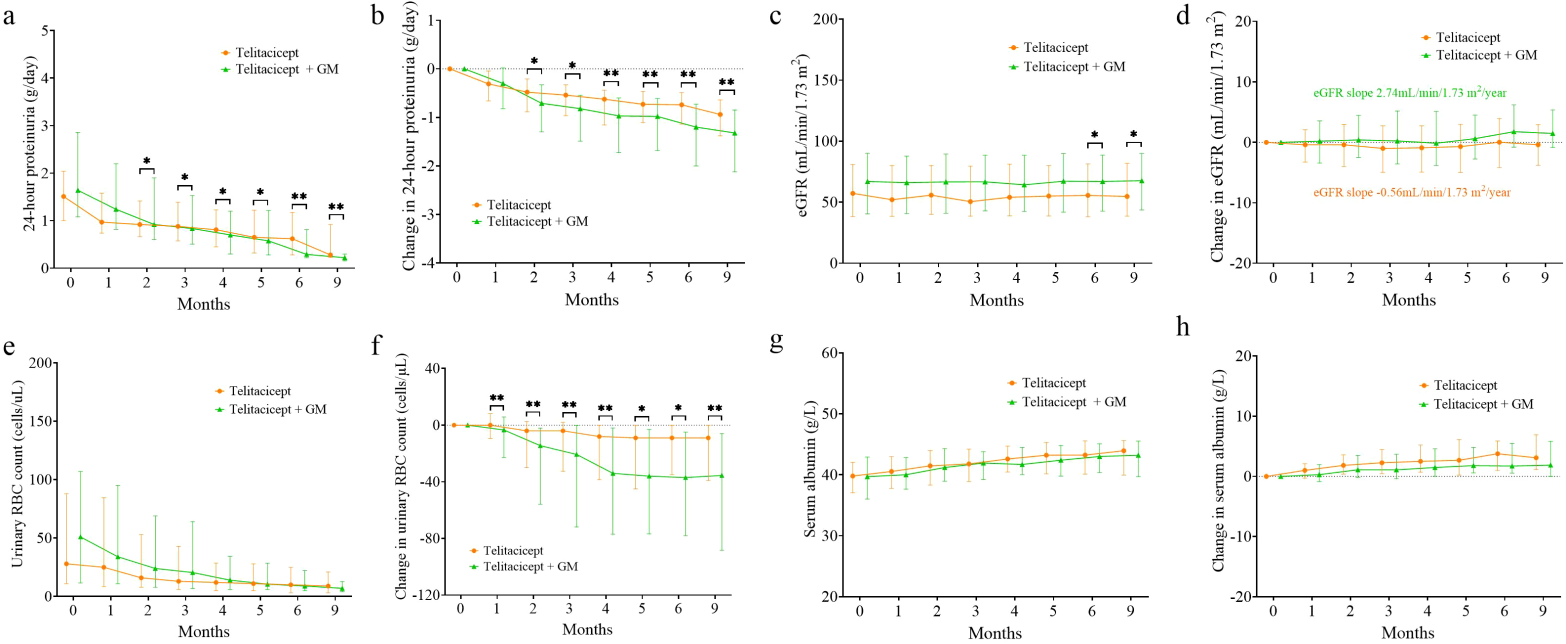
24-hour proteinuria, eGFR, urinary RBC count and serum albumin from baseline in the telitacicept group and the telitacicept + GM group at follow-up. **(a)** 24-hour proteinuria; **(b)** change in 24-hour proteinuria; **(c)** eGFR; **(d)** change in eGFR; **(e)** urinary RBC count; **(f)** change in urinary RBC count; **(g)** serum albumin; **(h)** change in serum albumin. The median and IQR are shown. *P < 0.05, ****P < 0.0001.

The level of 24-hour proteinuria at 9 months decreased from 1.64 g/day (IQR 1.08-2.86) to 0.22 g/day (IQR 0.16-0.31) in the telitacicept + GM group, and from 1.51 g/day (IQR 0.96-2.04) to 0.28 g/day (IQR 0.23–0.92) in the telitacicept group (**Figure 2a**). A significant difference in proteinuria between the groups emerged after 2 months of follow-up (**Figure 2a**). At 9 months, the change in proteinuria in the telitacicept + GM group was reduced to -1.32 g/day (IQR -0.85 to -2.12), a greater reduction than in the telitacicept group -0.94 g/day [(IQR -0.64 to -1.38), *P* < 0.001, **Figure 2b**]. In the telitacicept + GM group, a slight improvement in eGFR was observed at 9 months compared to baseline [67.6 ml/min/1.73 m^2^ (IQR 43.9-89.4) versus 66.9 ml/min/1.73 m^2^ (IQR 41.5-88.9), **Figure 2c**]. Conversely, the telitacicept group exhibited a decline in eGFR over the 9-month period [54.7 ml/min/1.73 m^2^ (IQR 38.6-81.5) versus 57.2 ml/min/1.73 m^2^ (IQR 38.3-80.8), **Figure 2c**]. This finding is supported by the eGFR slope of 2.74 ml/min/1.73 m^2^/year in the telitacicept + GM group, compared to -0.56 ml/min/1.73 m^2^/year in the telitacicept group (*P* = 0.014, **Figure 2d**).

Although the two groups showed comparable reductions in urinary RBC count over 9 months (**Figure 2e**), the magnitude of reduction was significantly greater in the telitacicept + GM group at the study endpoint, with a median change of -35.5/µL (IQR -88.0 to -6.0) compared to -9.0/µL (IQR -38.0 to 0.0) in the telitacicept group (*P* = 0.009; **Figure 2f**). Within 9 months of treatment, the changes in serum albumin levels were similar between the two groups (**Figures 2g, h**).

### 3.3 Secondary outcomes

**Figure 3:**
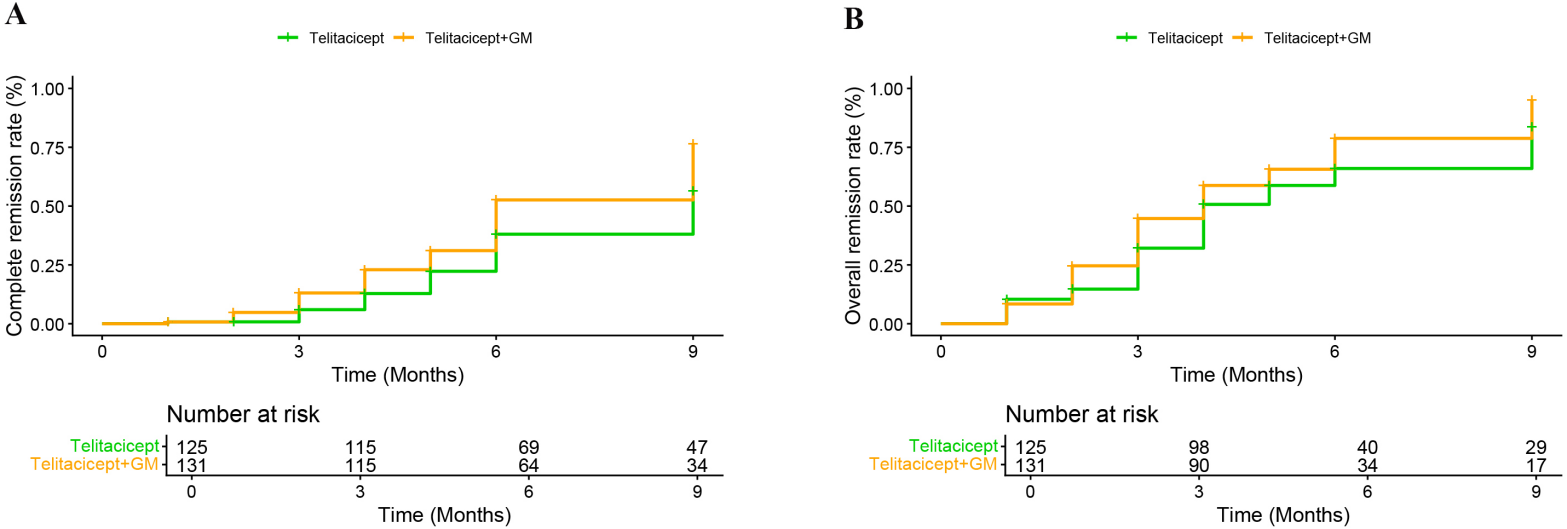
Cumulative probability of 9-month **(A)** complete remission (*log-rank P =* 0.005) and **(B)** overall remission (*log-rank P =* 0.014) using Kaplan-Meier analysis.

At 9 months, the CR and OR rates were 54.2% and 78.6% in the telitacicept + GM group, compared to 40.8% and 70.4% in the telitacicept group, respectively. Kaplan-Meier analysis confirmed that the telitacicept + GM group had significantly higher CR (log-rank test, χ^2^ = 7.829, *P* = 0.005) and OR (log-rank test, χ^2^ = 5.984, *P* = 0.014) rates over time (**Figures 3A, B**). We employed multivariable Cox proportional hazards models to evaluate the impact of treatment on 9-month CR and OR, with adjustments for various covariates across models (**Table 2**). In the crude model, the telitacicept + GM group was associated with a significantly increased likelihood of achieving both CR [HR 1.58 (95% CI 1.10-2.26), *P* = 0.013] and OR [HR 1.36 (95% CI 1.02-1.81), *P* = 0.035] rates at the 9-month follow-up compared to the telitacicept group. After sequential adjustment for age, sex, BMI, hypertension, proteinuria, urinary RBC count, eGFR, and Oxford classification scores, the fully adjusted model confirmed that the telitacicept + GM regimen was independently associated with higher CR [adjusted HR 2.18 (95% CI 1.46-3.22), *P* < 0.001] and OR [adjusted HR 1.46 (95% CI 1.08-2.00), *P* = 0.013] rates at the 9-month follow-up. A sensitivity analysis based on the complete-case dataset (excluding cases with missing pathology data) is presented in the **Supplementary Table S1**.

**Table 2:** Multivariable Cox proportional hazards model comparing the time to CR and OR between treatment regimens. .

Variable	Crude model		Model 1		Model 2		Model 3	
	HR (95% CI)	*P*-value	HR (95% CI)	*P*-value	HR (95% CI)	*P*-value	HR (95% CI)	*P*-value
CR								
Telitacicept	1 (Ref)		1 (Ref)		1 (Ref)		1 (Ref)	
Telitacicept + GM	1.58 (1.10-2.26)	0.013	1.65 (1.15-2.39)	0.007	2.17 (1.47-3.20)	<0.001	2.18 (1.46-3.22)	<0.001
OR								
Telitacicept	1 (Ref)		1 (Ref)		1 (Ref)		1 (Ref)	
Telitacicept + GM	1.36 (1.02-1.81)	0.035	1.37 (1.04-1.85)	0.028	1.48 (1.10-2.00)	0.009	1.46 (1.08-2.00)	0.013

Crude model: not adjusted; model 1, adjusted for age, sex, BMI; model 2, adjusted for model 1 plus hypertension, proteinuria, urinary RBC count and eGFR; model 3, adjusted for model 2 plus M, E, S, T, C scores.

Among the 256 participants, 136 were monitored for lymphocyte subsets and immunoglobulin levels at the 3-months point, with 69 in the telitacicept + GM group and 66 in the telitacicept group. At 3 months, there was a notable decrease in IgA, IgG, and IgM levels compared to the baseline (**Supplementary Figures S1a–c**). The median reduction in CD19+ B cells was 96.0 cells/µL (35.3%) in the telitacicept + GM group and 54.5 cells/µL (19.1%) in the telitacicept group at 3 months, with both reductions being statistically significant from baseline (**Supplementary Figure S1d**). In contrast, CD4+ T cells decreased significantly only in the telitacicept+GM group at 3 months (**Supplementary Figure S1f**). Serum complement C3 levels remained stable in both groups (**Supplementary Figure S1g**).

### 3.4 Subgroup analysis

**Figure 4:**
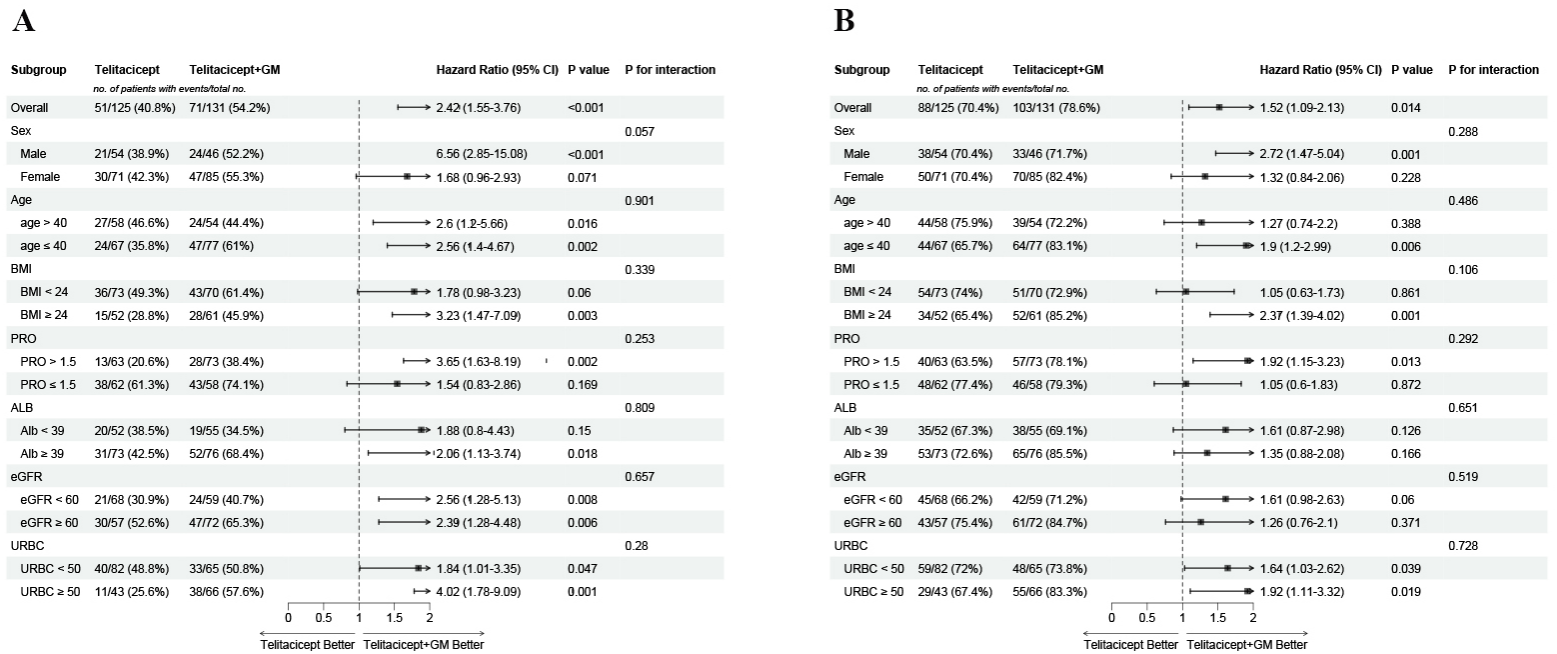
Subgroup analyses of the association between treatment regimen and 9-month **(A)** complete remission and **(B)** overall remission.

Subgroup analysis revealed consistent benefits of the telitacicept + GM regimen across all predefined subgroups, with no significant interaction effects detected (all *P* for interaction ≥ 0.05), indicating that the treatment effects of telitacicept + GM were consistent regardless of baseline characteristics such as age, sex, eGFR, or proteinuria (**Figures 4A, B**).

The telitacicept + GM group was further stratified into two subgroups: telitacicept + glucocorticoids (n = 46) and telitacicept + MMF (n = 69) (**Figure 1**), which were well-balanced at baseline (**Supplementary Table S2**). With the exception of between-group differences in the reduction of hematuria levels at 2–4 months, no significant differences were observed between the two subgroups in terms of proteinuria, eGFR, or serum albumin levels (**Supplementary Figure S2**). Consistent with these findings, Kaplan-Meier analysis revealed no significant between-subgroup differences in either CR or OR rate (**Supplementary Figure S3**).

### 3.5 Safety and adverse events

The telitacicept + GM group experienced a greater overall rate of non-serious side effects, such as obesity, osteoporosis, gastritis, and fatigue, compared to the telitacicept group (28.2% versus 11.2%, **Supplementary Table S3**), but neither treatment group reported any serious adverse events.

## 4 Discussion

This multicenter, retrospective study represents the first real-world comparison of telitacicept combining immunosuppressants therapy versus telitacicept monotherapy in IgAN patients. Our findings suggest that the telitacicept plus GM therapy is associated with significant reductions in proteinuria and hematuria, as well as slightly higher eGFR levels at the 9-month mark. Additionally, the telitacicept plus GM therapy is correlated with significantly higher rates of CR and OR, without the occurrence of serious adverse reactions. These results suggest that telitacicept plus GM therapy may provide superior renal protection compared to telitacicept monotherapy.

Current research has demonstrated that BAFF and APRIL promote the production of galactose-deficient IgA1 by B cells and facilitate the generation of mucosal plasma cells (21). Phase II clinical research showed that combined BAFF and APRIL inhibitors (atacicept, EudraCT 2020-004892-41, and telitacicept, NCT05596708) decreased proteinuria in patients with IgAN (11, 22). A large retrospective study conducted across 19 centers in China further revealed that telitacicept significantly and safely reduced proteinuria in patients with IgAN (23). He et al. (24) reported that proteinuria levels in the telitacicept group were decreased from a baseline of 1.6 g/day to 0.5 g/day, compared to a reduction from 1.7 g/day to 0.8 g/day in the MMF group over 6 months. Although the KDIGO 2025 guidelines (17) recommend a proteinuria target of less than 0.3 g/day, the lack of established optimal strategies means this goal remains challenging to attain with currently available therapies. Therefore, combining telitacicept with immunosuppressants is necessary to reduce proteinuria.

Our prior research indicated comparable efficacy in proteinuria reduction between half-dose corticosteroids combined with renin-angiotensin system blockers and full-dose corticosteroids alone (25). MMF has demonstrated heightened efficacy in suppressing B cell antibody production, studies have showed that MMF alone or combined with corticosteroids is beneficial for patients with progressive IgAN (18, 26). The effectiveness of combination therapy with telitacicept for IgAN remains a topic of ongoing research, with varying conclusions reported across different studies. In a recent 24-month follow-up study, telitacicept plus low-dose MMF was shown to decrease proteinuria by 3.99 g/day (87% from baseline) while maintaining stable eGFR levels (27). Additionally, a retrospective real-world study involving 11 patients indicated that a low-to-medium dose of telitacicept, when combined with conventional therapy, significantly reduced proteinuria by 64.38% and increased eGFR by 23.18% at 24 weeks (28). Liu et al. (23) conducted a subgroup analysis which revealed comparable proteinuria reduction throughout the study duration [0.6 g/day (0.1, 1.5) vs. 0.8 g/day (0.1, 2.0)] between the telitacicept group and the telitacicept combined with glucocorticoid/immunosuppressor group. In our investigation, the telitacicept plus GM group showed a significantly greater reduction in proteinuria (-1.32 g/day versus -0.94 g/day) and exhibited better eGFR slope (2.74 ml/min/1.73 m^2^/year versus -0.56 ml/min/1.73 m^2^/year) compared to the telitacicept group. This finding may be explained by a synergistic effect whereby the therapy both switches off the production of pathogenic IgA and rapidly halts glomerular inflammation, thus preserving long-term kidney function (29). Hematuria was significantly alleviated alongside the reduction in proteinuria in the telitacicept plus GM group, with a median change of -35.5/µL (IQR -88.3 to -6.0). This observed relief in hematuria is clinically meaningful, as it represents another key indicator of disease progression in IgAN (30). Furthermore, multivariable analysis indicated that telitacicept plus GM therapy was associated with a higher likelihood of achieving 9-month CR [adjusted HR 2.58 (95% CI 1.65-4.04)] and OR [adjusted HR 1.67 (95% CI 1.18-2.37)] rates. As previously observed in IgAVN patients (31), combining telitacicept with immunosuppressive therapy led to notable enhancements in CR rates and the stabilization of renal function.

The serum immunoglobulin levels (including IgA, IgG and IgM) were significantly reduced at 3 months in both groups, aligning with results from the phase 2 clinical trial (32). Infections occurred in 6 (5.3%) patients in the telitacicept group and 12 (10.4%) in the telitacicept plus GM group; all infections were mild and did not necessitate hospitalization. The trend toward more infections in the telitacicept plus GM group may be attributed to a more substantial reduction in CD4^+^ T cells. The median reduction in CD19^+^ B cells was 19.1% in the telitacicept group and 35.3% in the telitacicept plus GM group. Notably, the clinical relevance of B-cell depletion appears to differ by disease: while membranous nephropathy necessitates extensive CD19^+^ B-cell depletion for remission (33), IgAN can achieve remission with only a moderate reduction. This observation suggests that the role of pathogenic B cells may vary according to their developmental stages, functional subsets, or anatomical niches across different nephropathies (34).

In terms of safety, the telitacicept plus GM group exhibited a higher incidence of conditions such as obesity, osteoporosis, gastritis, and fatigue, which were not observed in the telitacicept monotherapy group. Consequently, the telitacicept plus GM therapy was associated with a higher overall incidence of adverse events (28.2% vs. 11.2%). Nevertheless, both therapeutic approaches were with no serious adverse events occurring during the study.

This study has several limitations. Firstly, this investigation was a multicenter, retrospective, observational study. The assignment to treatment groups was non-randomized and based on clinical judgments, which could have been influenced by factors such as disease severity, physician preference, patient choice, and other variables, potentially introducing unmeasured confounding. Secondly, the study cohort was relatively homogeneous, comprising only Chinese patients receiving telitacicept at a dosage of 160 mg, which may restrict the generalizability of the findings to other populations. Thirdly, although adjustments were made for known confounders, the loss to follow-up could have skewed the results towards outcomes in patients who tolerated and adhered to the treatment regimen. Future studies should prioritize prospective, randomized controlled trials that include ethnically diverse cohorts, extend the follow-up period, and incorporate comprehensive immunological profiling to better elucidate the long-term efficacy differences between telitacicept plus glucocorticoids and telitacicept plus MMF in IgAN.

In conclusion, the combination therapy of telitacicept with immunosuppressants may represent an effective treatment approach for IgAN patients by reducing proteinuria, improving response rates, preserving eGFR, and maintaining a favorable safety profile without serious adverse events.

## Data Availability

The original contributions presented in the study are included in the article/**Supplementary Material**. Further inquiries can be directed to the corresponding authors.

## Supplementary material

Supplementary Table 1 Multivariable Cox proportional hazards model comparing the time to CR and OR between treatment regimens. Crude model: not adjusted; model 1: adjusted for age, sex, BMI;model 2: adjusted for model 1 plus hypertension, proteinuria, urinary RBC count and eGFR;model 3: adjusted for model 2 plus M, E, S, T, C scores (based on the complete-case dataset).

Supplementary Table 2 Baseline clinical characteristics of patients in the telitacicept + GM group. ^a^ Oxford classification and Lee’s classification was collected from 101 patients; G, glucocorticoid; M, mycophenolate mofetil; BMI, body mass index; MBP, mean blood pressure; DBP, diastolic blood pressure; RBC, red blood cells; eGFR, estimated glomerular filtration rate; ALT, alanine aminotransferase; AST, aspartate aminotransferase; TC, total cholesterol; TG, triglyceride; M, mesangial hypercellularity; E, endocapillary hypercellularity; S, segmental glomerulosclerosis; T, tubular atrophy/interstitial fibrosis; C, crescents.

Supplementary Table 3 Summary of adverse events. AE, adverse event.

Supplementary Figure 1 Serum IgA, IgG, IgM, CD19+ B cells, CD 4+ T cells and C3 levels between baseline and 3 months after treatment.

Supplementary Figure 2 Subgroup analyses of changes from baseline to follow-up: 24-hour proteinuria, eGFR, urinary RBC count, and serum albumin (Telitacicept + G vs. Telitacicept + M). **(a)** 24-hour proteinuria; **(b)** change in 24-hour proteinuria; **(c)** eGFR; **(d)** change in eGFR; **(e**) urinary RBC count; **(f)** change in urinary RBC count; **(g)** serum albumin; **(h)** change in serum albumin. G, glucocorticoid; M, mycophenolate mofetil; The median and IQR are shown. *P < 0.05.

Supplementary Figure 3 Subgroup analyses of cumulative probability of 9-month **(A)** complete remission and **(B)** overall remission using Kaplan-Meier analysis. G, glucocorticoid; M, mycophenolate mofetil.
