# Supplementary material for: Efficacy and safety of telitacicept combined with immunosuppressive therapy for IgA nephropathy: a retrospective multicenter cohort study
Source: Front Immunol. 2026 Feb 11;17:1740891. doi: 10.3389/fimmu.2026.1740891 (PMC12932424; doi:10.3389/fimmu.2026.1740891)
Supplement: Supplementary Table 1 — Multivariable Cox proportional hazards model comparing the time to CR and OR between treatment regimens. Crude model: not adjusted; model 1: adjusted for age, sex, BMI;model 2: adjusted for model 1 plus hypertension, proteinuria, urinary RBC count and eGFR;model 3: adjusted for model 2 plus M, E, S, T, C scores (based on the complete-case dataset). [file Table1.docx]

**Supplementary Table 1: Multivariable Cox proportional hazards model comparing the time to CR and OR between treatment regimens.**

|  | Crude model | |  | Model 1 | |  | Model 2 | |  | Model 3 | |
| --- | --- | --- | --- | --- | --- | --- | --- | --- | --- | --- | --- |
| Variable | HR (95% CI) | *P*-value |  | HR (95% CI) | *P*-value |  | HR (95% CI) | *P*-value |  | HR (95% CI) | *P*-value |
| CR |  |  |  |  |  |  |  |  |  |  |  |
| Telitacicept | 1 (Ref) |  |  | 1 (Ref) |  |  | 1 (Ref) |  |  | 1 (Ref) |  |
| Telitacicept + GM | 1.58 (1.10-2.26) | 0.013 |  | 1.65 (1.15-2.39) | 0.007 |  | 2.17 (1.47-3.20) | <0.001 |  | 2.17 (1.46-3.22) | <0.001 |
| OR |  |  |  |  |  |  |  |  |  |  |  |
| Telitacicept | 1 (Ref) |  |  | 1 (Ref) |  |  | 1 (Ref) |  |  | 1 (Ref) |  |
| Telitacicept + GM | 1.36 (1.02-1.81) | 0.035 |  | 1.37 (1.04-1.85) | 0.028 |  | 1.48 (1.10-2.00) | 0.009 |  | 1.46 (1.05-2.05) | 0.024 |

Crude model: not adjusted;
model 1: adjusted for age, sex, BMI;
model 2: adjusted for model 1 plus hypertension, proteinuria, urinary RBC count and eGFR;
model 3: adjusted for model 2 plus M, E, S, T, C scores (based on the complete-case dataset).

**Supplementary Table 2: Baseline clinical characteristics of patients in the telitacicept + GM group.**

| Variables | Telitacicept + G  (n = 46) | | Telitacicept + M  (n = 69) | *P*-value |
| --- | --- | --- | --- | --- |
| Age (years) | 39.7 ± 11.7 | | 38.0 ± 12.0 | 0.923 |
| Male, n (%) | 16 (34.8) | | 22 (31.9) | 0.746 |
| BMI (kg/m^2^) | 23.1 ± 3.9 | | 24.2 ± 3.8 | 0.884 |
| Hypertension, n (%) | 22 (47.8) | | 34 (49.3) | 0.879 |
| SBP | 130.4 ± 16.9 | | 133.9 ± 20.8 | 0.170 |
| DBP | 83.9 ± 11.6 | | 86.7 ± 16.5 | 0.060 |
| 24-hour proteinuria (g/day) | 1.5 (1.0, 2.9) | | 1.2 (1.0, 2.1) | 0.211 |
| Urinary RBC count (cells/uL) | 34.5 (9.1, 132.5) | | 25.0 (3.5, 129.0) | 0.151 |
| Hemoglobin (g/L) | 125.0 ± 20.2 | | 127.7 ± 19.6 | 0.603 |
| Serum albumin (g/L) | 38.8 ± 5.4 | | 40.4 ± 5.0 | 0.518 |
| Urea creatinine (μmol/L) | 123.0 ± 67.2 | | 114.2 ± 69.8 | 0.973 |
| eGFR (mL/min/1.73 m^2^) | 67.1 (41.0, 84.8) | | 74.0 (39.6, 90.9) | 0.348 |
| Serum uric acid (µmol/L) | 382.6 ± 102.3 | | 389.6 ± 110.3 | 0.641 |
| ALT (U/L) | 23.4 ± 20.9 | | 22.6 ± 20.4 | 0.618 |
| AST (U/L) | 22.1 ± 9.6 | | 22.6 ± 14.3 | 0.589 |
| TC (mmol/L) | 5.3 ± 1.3 | | 5.3 ± 1.3 | 0.819 |
| TG (mmol/L) | 2.0 ± 1.5 | | 2.3 ± 1.8 | 0.216 |
| Glucose (mmol/L) | 5.2 ± 1.1 | | 5.5 ± 1.2 | 0.843 |
| Serum IgG (g/L) | 12.9 (10.2, 14.3) | | 12.1 (9.5, 14.7) | 0.454 |
| Serum IgA (g/L) | 3.1 (2.1, 4.7) | | 2.9 (2.3, 3.8) | 0.751 |
| Serum IgM (g/L) | 1.5 (1.1, 1.6) | | 1.2 (0.9, 1.7) | 0.570 |
| Oxford classification ^a^ | |  |  |  |
| M1 | 39 (95.1) | | 56 (93.3) | 0.709 |
| E1 | 12 (29.3) | | 20 (33.3) | 0.666 |
| S1 | 30 (73.2) | | 39 (65.0) | 0.386 |
| T 1/2 | 22 (53.7) | | 25 (41.7) | 0.235 |
| C 1/2 | 22 (53.7) | | 27 (45.0) | 0.393 |
| Lee’s classification ^a^ |  | |  | 0.872 |
| II | 4 (9.8) | | 7 (11.7) |  |
| III | 21 (51.2) | | 27 (45.0) |  |
| IV | 14 (34.1) | | 21 (35.0) |  |
| V | 2 (4.9) | | 5 (8.3) |  |

^a^ Oxford classification and Lee’s classification was collected from 101 patients; G, glucocorticoid; M, mycophenolate mofetil; BMI, body mass index; MBP, mean blood pressure; DBP, diastolic blood pressure; RBC, red blood cells; eGFR, estimated glomerular filtration rate; ALT, alanine aminotransferase; AST, aspartate aminotransferase; TC, total cholesterol; TG, triglyceride; M, mesangial hypercellularity; E, endocapillary hypercellularity; S, segmental glomerulosclerosis; T, tubular atrophy/interstitial fibrosis; C, crescents.

**Supplementary Table 3: Summary of adverse events.**

| Events | Telitacicept + GM (n = 131) | Telitacicept (n = 125) |
| --- | --- | --- |
| Incidence of AEs | 37 (28.2) | 14 (11.2) |
| Injection site reactions | 10 (7.6) | 8 (6.4) |
| Infection | 12 (9.2) | 6 (4.8) |
| Skin acne | 1 (0.8) | 0 (0.0) |
| Obesity | 6 (4.6) | 0 (0.0) |
| Fatigue | 1 (0.8) | 0 (0.0) |
| Osteoporosis | 4 (3.1) | 0 (0.0) |
| Gastroenteritis | 2 (1.5) | 0 (0.0) |
| Vision impairment | 1 (0.8) | 0 (0.0) |

AE, adverse event.
