# Supplementary figures and images for: Efficacy and safety of telitacicept combined with immunosuppressive therapy for IgA nephropathy: a retrospective multicenter cohort study
Source: Front Immunol. 2026 Feb 11;17:1740891. doi: 10.3389/fimmu.2026.1740891 (PMC12932424; doi:10.3389/fimmu.2026.1740891)

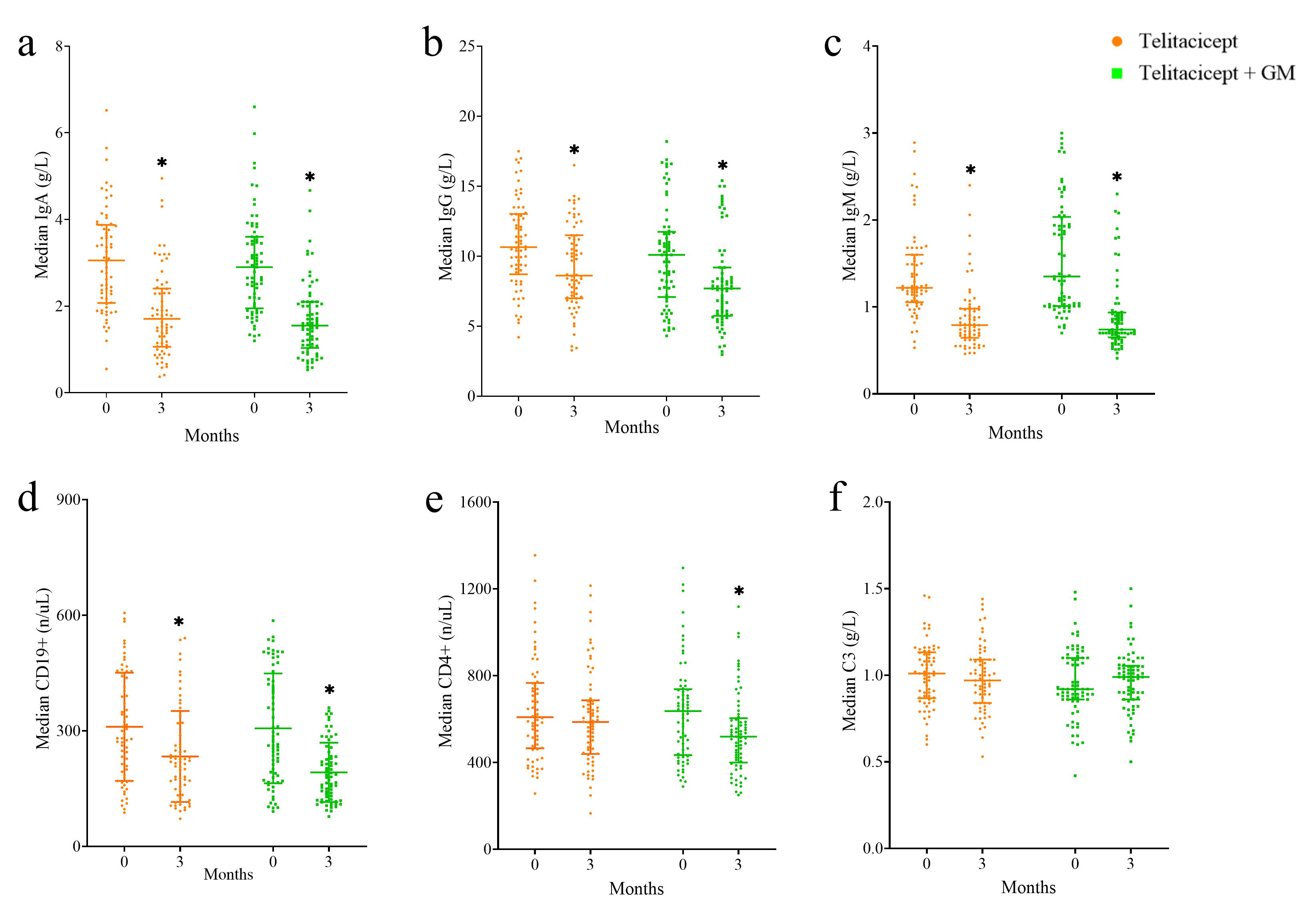

Supplement: Supplementary Figure 1 — Serum IgA, IgG, IgM, CD19+ B cells, CD 4+ T cells and C3 levels between baseline and 3 months after treatment. [file Image1.jpeg]

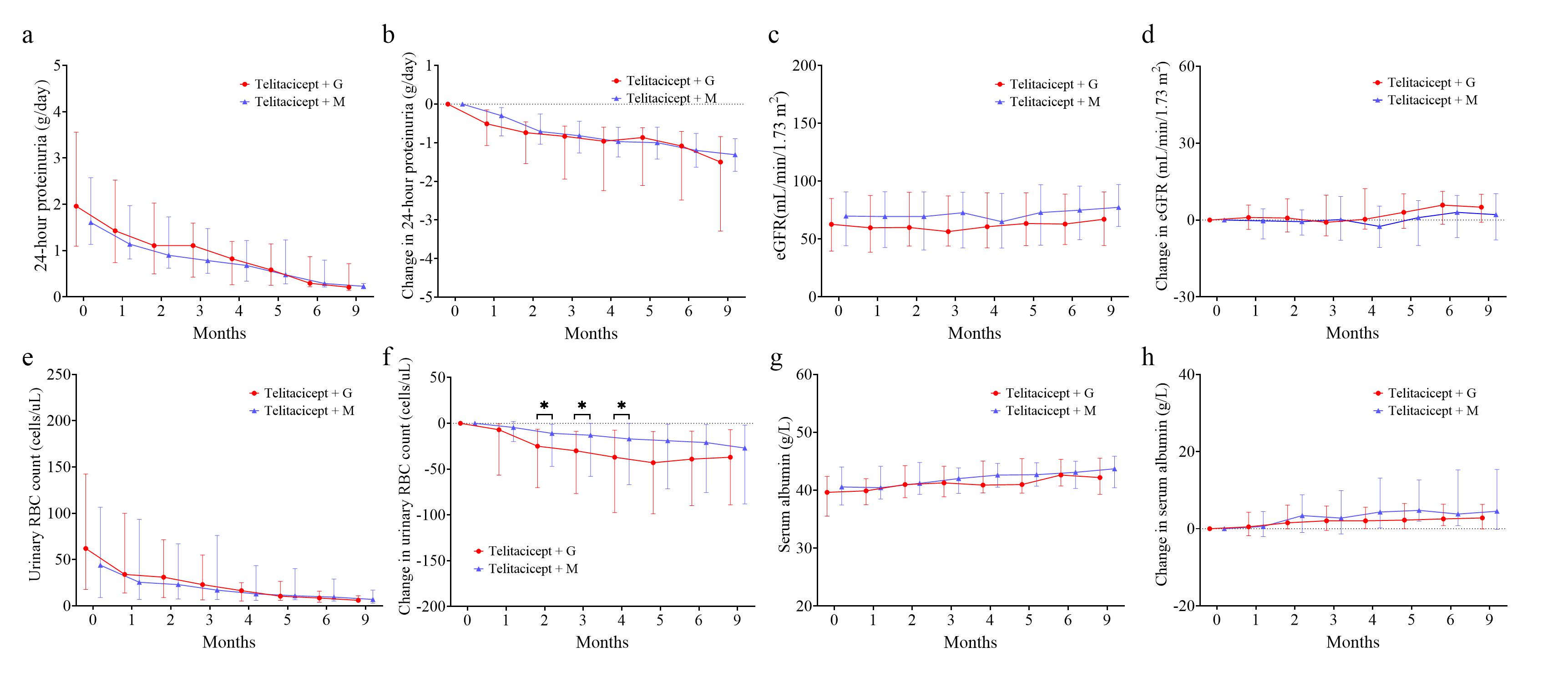

Supplement: Supplementary Figure 2 — Subgroup analyses of changes from baseline to follow-up: 24-hour proteinuria, eGFR, urinary RBC count, and serum albumin (Telitacicept + G vs. Telitacicept + M). (a) 24-hour proteinuria; (b) change in 24-hour proteinuria; (c) eGFR; (d) change in eGFR; (e) urinary RBC count; (f) change in urinary RBC count; (g) serum albumin; (h) change in serum albumin. G, glucocorticoid; M, mycophenolate mofetil; The median and IQR are shown. *P < 0.05. [file Image2.jpeg]

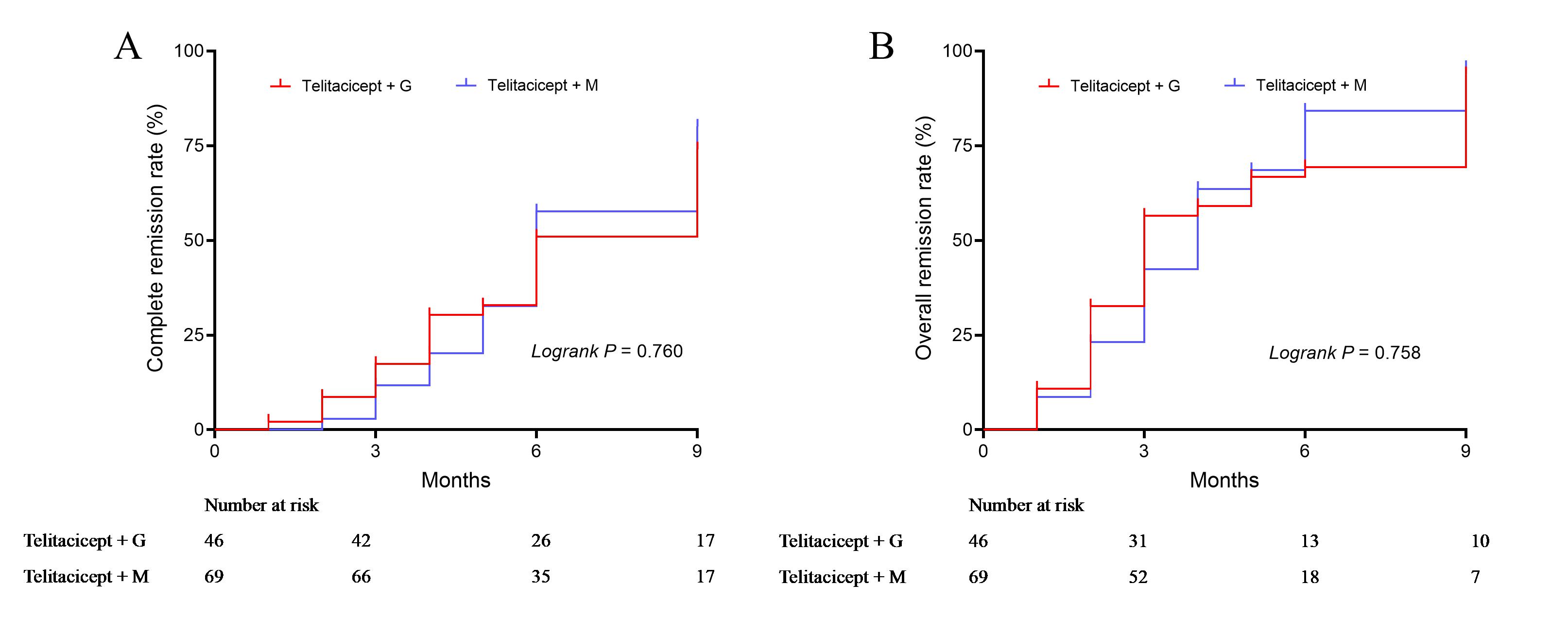

Supplement: Supplementary Figure 3 — Subgroup analyses of cumulative probability of 9-month (A) complete remission and (B) overall remission using Kaplan-Meier analysis. G, glucocorticoid; M, mycophenolate mofetil. [file Image3.jpeg]
